# Age Influences Microglial Activation After Cuprizone-Induced Demyelination

**DOI:** 10.3389/fnagi.2018.00278

**Published:** 2018-09-20

**Authors:** Barbara Klein, Heike Mrowetz, Conor Michael Barker, Simona Lange, Francisco J. Rivera, Ludwig Aigner

**Affiliations:** ^1^Institute of Molecular Regenerative Medicine, Paracelsus Medical University, Salzburg, Austria; ^2^Spinal Cord Injury and Tissue Regeneration Center Salzburg (SCI-TReCS), Paracelsus Medical University, Salzburg, Austria; ^3^Laboratory of Stem Cells and Neuroregeneration, Institute of Anatomy, Histology and Pathology, Faculty of Medicine, Universidad Austral de Chile, Valdivia, Chile; ^4^Center for Interdisciplinary Studies on the Nervous System (CISNe), Universidad Austral de Chile, Valdivia, Chile

**Keywords:** microglia, aging, demyelination, cuprizone, hippocampus, corpus callosum, TSPO, P2RY12

## Abstract

Multiple sclerosis (MS) is a chronic inflammatory CNS disease, which causes demyelinated lesions and damages white and gray matter regions. Aging is a significant factor in the progression of MS, and microglia, the immune cells of the CNS tissue, play an important role in all disease stages. During aging, microglia are functionally altered. These age-related changes probably already begin early and might influence the progression of CNS pathologies. The aim of the present study was to investigate whether microglia in the middle-aged CNS already react differently to demyelination. For this purpose, several microglia markers (ionized calcium binding adaptor molecule 1 (Iba-1), P2RY12, F4/80, CD68, major histocompatibility complex II (MHCII), macrophage receptor with collagenous structure (Marco), Translocator protein 18 kD (TSPO), CD206, and CD163) were analyzed in the acute cuprizone demyelination model in young (2-month-old) and middle-aged (10-month-old) mice. In addition, microglial proliferation was quantified using double-labeling with proliferating cell nuclear antigen (PCNA) and bromodeoxyuridine (BrdU), which was injected with the onset of remyelination. To compare age-related microglial changes during de- and remyelination in both gray and white matter, the hilus of the dorsal hippocampal dentate gyrus (DG) and the splenium of the corpus callosum (CC) were analyzed in parallel. Age-related changes in microglia of healthy controls were more pronounced in the analyzed gray matter region (higher levels of F4/80 and Marco as well as lower expression of CD68 in middle-aged mice). During de- and remyelination, a stronger increase of the microglial markers Iba-1, CD68 and TSPO was observed in the splenium of the younger groups. There was a significant reduction of P2RY12 during demyelination, however, this was age- and region-dependent. The induction of the anti-inflammatory markers CD206 and CD163 was stronger in the middle-aged group, but also differed between the two analyzed regions. De- and remyelination led to a significant increase in PCNA^+^ microglia only in young groups within the white matter region. The number of BrdU^+^ microglia was not changed during de- or remyelination. These results clearly show that microglia are already altered during middle-age and also react differently to CNS demyelination, however, this is highly region-dependent.

## Introduction

With increasing age, microglia, the resident immune cells of the CNS parenchyma, undergo functional changes (reviewed in Mosher and Wyss-Coray, 2014). Aging microglia seem to become more reactive to pro-inflammatory stimuli, and at the same time, also more resistant to deactivation, which together lead to an exaggerated and longer lasting inflammatory response. This process has been described as “microglial priming” (reviewed in Norden and Godbout, 2013; Norden et al., 2015), and may influence the progression of CNS pathologies (reviewed in Perry and Holmes, 2014). Additionally, there is evidence that microglia functions (such as phagocytosis and proteostasis), which are important for CNS homeostasis and in reaction to CNS pathologies, become impaired during aging (reviewed in Mosher and Wyss-Coray, 2014). These age-related microglial changes most likely already start during adulthood (e.g., Bardou et al., 2013; Lee et al., 2013; Hefendehl et al., 2014), until, at extreme ages, microglia become senescent and dystrophic (reviewed in Streit et al., 2014). The exact time sequence and functional impact of microglial age-related changes, however, need to be studied more extensively.

Multiple sclerosis (MS) is a chronic inflammatory disease of the CNS, which causes demyelination not only in white matter regions, but also damages gray matter and leads to axonal loss and neurodegeneration (reviews of MS pathology: Mallucci et al., 2015; Baecher-Allan et al., 2018). In young adults, MS is the main cause of non-traumatic neurological disabilities (Browne et al., 2014). Despite different MS disease patterns, aging is a very important factor in the progression of MS pathology and increasing disability (Confavreux and Vukusic, 2006; Manouchehrinia et al., 2017).

Microglia accumulate in active MS lesions and play an important role in all stages of MS pathology (reviewed in Jack et al., 2005; Rawji and Yong, 2013; O’Loughlin et al., 2018). There are indications that microglia can facilitate myelin repair by phagocytosing myelin and cell debris, secreting pro-regenerative signals (e.g., growth factors and cytokines), and by influencing oligodendrocyte lineage cells (reviewed in Napoli and Neumann, 2010; McMurran et al., 2016; Miron, 2017). However, with increasing disease duration and age, myelin regeneration becomes less successful, and it is possible that age-related changes in microglia are one factor contributing to this failure in myelin repair. Even though this hypothesis is not new, most preclinical studies on microglia in models for MS or demyelination used animals of a young age, which could conceivably influence the results.

The aim of the present study was to investigate whether microglia in the middle-aged (10-months-old) CNS react differently to demyelination than young microglia (2-months-old). For this purpose, we performed a comprehensive analysis of microglia using cell- and activity-specific markers in combination with markers for proliferation in the acute cuprizone demyelination model. This model is a well-studied and reproducible toxic model, which induces demyelination followed by spontaneous remyelination (Kipp et al., 2009; Praet et al., 2014; Tagge et al., 2016). Similar to the pathology in human MS patients, in the cuprizone model demyelination and axonal damage not only occur in white matter regions, like the corpus callosum (CC), but also in gray matter (Goldberg et al., 2015), for example, in the hippocampus (Hoffmann et al., 2008; Koutsoudaki et al., 2009; Norkute et al., 2009; Sun et al., 2016). To compare age-related microglial changes in both gray and white matter after demyelination, the hilus of the hippocampal dentate gyrus (DG) and the splenium of the CC were analyzed in parallel. The hippocampus was chosen as a target of our investigation, since MS patients often suffer from episodic memory impairment, which might be caused by pathological changes (such as altered connectivity, microstructural damage, atrophy and demyelination) in this region (Geurts et al., 2007; Sicotte et al., 2008; Dutta et al., 2011; Hulst et al., 2015; Planche et al., 2017). Recently, it has been shown that the CA4/DG subfield is the first region of the hippocampus that is atrophied at the earliest stages of MS (Planche et al., 2018). While studies about aging microglia often focus on changes affecting the old CNS, we specifically chose to analyze middle-aged—not old—animals, since in MS patients also around middle age, regeneration starts to fail and irreversible disabilities occur (Confavreux and Vukusic, 2006; Manouchehrinia et al., 2017). The results of this analysis should provide insight into the extent of age-related changes in middle-aged microglia, which could be a useful background for other studies, for example, to test new treatment strategies to enhance remyelination.

Studies about microglia largely depend on antibodies that recognize microglia activity state-specific antigens. This is an ongoing topic of research, and the list of useful markers and antibodies is constantly growing. Two markers that are strongly expressed in microglia surveying the healthy CNS, and thus, are ideal for general morphological analyses, are: (i) ionized calcium binding adaptor molecule 1 (Iba-1), which is (like most conventionally used microglia markers) expressed by microglia and infiltrating macrophages, and plays a role in calcium homeostasis (Imai et al., 1996; Ito et al., 1998); and (ii) the G-protein-coupled purinergic receptor P2RY12, which distinguishes microglia from CNS-infiltrating monocytes and other peripheral macrophages (Sasaki et al., 2003; Butovsky et al., 2014; Bennett et al., 2016). Despite their use as general markers for microglia, the expression of Iba-1 and P2RY12 is differentially affected by inflammatory stimuli, which lead to an up-regulation of Iba-1 (e.g., Mori et al., 2000; Ito et al., 2001), whereas P2RY12 is increased during anti-inflammatory conditions (e.g., Moore et al., 2015; Beaino et al., 2017). Also F4/80, an adhesion G protein-coupled receptor that is expressed in mature mouse macrophages and microglia and may be involved in the induction of peripheral immune tolerance, is often analyzed (reviewed in Gordon et al., 2011). In contrast to Iba-1 and P2RY12, F4/80 is expressed at relatively low levels in microglia. One of the most widely used markers for microglial activation, CD68 (also ED1 or macrosialin), is a lysosomal/endosomal-associated protein, that plays a role in microglial phagocytosis (Fulci et al., 2007), however, its exact function is still unknown (reviewed in Sierra et al., 2013). Another popular microglia activation marker is major histocompatibility complex II (MHCII). In contrast to professional antigen-presenting cells, the expression of MHCII is low in microglia in the healthy CNS, however, it can be upregulated upon activation (reviewed in Sierra et al., 2013). The macrophage receptor with collagenous structure (Marco) is a class A scavenger receptor that has been associated with a pro-inflammatory microglial activation (reviewed in Colton, 2009; Sierra et al., 2013), and plays a role in defense against bacterial meningitis (Braun et al., 2011) and in binding amyloid-β (Brandenburg et al., 2010). Translocator protein 18 kD (TSPO), another pro-inflammatory marker, is involved in the transport of cholesterol across mitochondrial membranes, and is increased in neuroinflammatory diseases in microglia and astrocytes. TSPO is of particular interest, since it is used as a target for PET imaging, for example with the tracer PK11195 (reviewed in Jacobs and Tavitian, 2012). This PET signal is increased in healthy aged humans (Schuitemaker et al., 2012), and was also shown to correlate in MS patients with age, disease progression and clinical outcome (reviewed in Airas et al., 2018). Two markers that typically used to identify anti-inflammatory microglia are the mannose receptor CD206, which is not only expressed in microglia, but also in astrocytes (Burudi et al., 1999; Régnier-Vigouroux, 2003) and plays a role in the phagocytosis of pathogens (reviewed in Sierra et al., 2013), and the hemoglobin-haptoglobin scavenger receptor CD163, which is expressed in microglia and perivascular macrophages (reviewed in Colton, 2009; Thomsen et al., 2013). Proliferating microglia are often identified using co-labeling with proliferating cell nuclear antigen (PCNA). In addition, the generation and subsequent survival and fate of microglia can be detected after labeling proliferating cells *in vivo* using for example bromodeoxyuridine (BrdU).

## Materials and Methods

### Animals and Cuprizone Treatment

Male C57BL/6J mice, at the ages of 2 and 10 months, were obtained from Janvier and afterwards kept in the animal facility of the Paracelsus Medical University in Salzburg, Austria under standard animal housing conditions with food and water *ad libitum*. Mice were divided into four different treatment groups per age, with six animals in each group, resulting in a total number of 48 animals. Control animals received a diet of standard food pellets (ssniff Spezialdiäten) for 8 weeks. For demyelination, animals in the respective groups received standard food pellets pre-mixed with 0.2% cuprizone (ssniff Spezialdiäten) for 6 weeks. To allow for remyelination, afterwards a normal diet consisting of standard food pellets was resumed for 1 or 2 weeks. For the analysis of proliferation of microglia and their subsequent survival during the remyelination period, animals received i.p. injections of BrdU (50 mg/kg body weight, in PBS) on six consecutive days starting on the first day after cuprizone administration. All experimental procedures were approved by the Federal State Government Salzburg, Austria (permit 20901-TVG/61/14-2016) and carried out in compliance with international ethical guidelines.

The percentage of weight change over the course of the experiment was calculated for each animal by subtracting the mean weight on the 2 days before the start of cuprizone treatment from the mean weight on the last 2 days before tissue collection.

### Tissue Preparation

Perfusion fixation and tissue preparation were done as previously described (Couillard-Despres et al., 2005). In short, mice were terminally anesthetized by injection (i.p.) of ketamine (273 mg/kg body weight), xylazine (71 mg/kg body weight) and acepromazine (4 mg/kg body weight) in a physiological NaCl solution and then transcardially perfused with a 0.9% NaCl (w/v) solution followed by phosphate-buffered 4% paraformaldehyde (pH 7.4). Afterwards, brains were extracted, postfixed overnight in 4% paraformaldehyde, cryoprotected in 30% sucrose (w/v in PBS), and sectioned on dry ice with a sliding microtome (Leica SM 2000R). For quantitative analysis, a representative tenth of one brain hemisphere was collected (i.e., every tenth section, with an interval of 400 μm between sections). The 40 μm-thick sections were stored at −20°C in a cryoprotection solution (equal parts glycerin, 0.2 M phosphate buffer, ethylene glycol and H_2_O).

### Immunohistochemistry

Immunohistological stainings were done according to a protocol that was previously described in detail (Kandasamy et al., 2014), with the addition of an antigen retrieval step after the first washing of the tissue sections. In this step, the sections were incubated in 10 mM citrate buffer (pH 6.0) in a steamer at 100°C for 20 min, followed by washing in PBS for 3 × 5 min. For two antibodies, CD163 and CD206, the antigen retrieval was shortened, i.e., the hot citrate buffer was put on the sections only for 10 min at RT. For each staining, a tenth of a hemisphere was used for immunohistochemistry.

The following antibodies and dilutions were used: primary antibodies: rat anti-myelin basic protein (anti-MBP; 1:150, MCA409S, AbD Serotec), goat anti-Iba-1 (1:500, Ab107159, Abcam), rabbit anti-P2RY12 (1:500, 55043A, AnaSpec), rabbit anti-CD68 (1:500, AB125212, Abcam), rat anti-MHCII (1:100, 14-5321-82, eBioscience), rat anti-Marco (1:200, MCA1849, AbD Serotec), rabbit anti-TSPO (PBR; 1:250, Novus Biologicals, NBP1-95674), mouse anti-CD206 (1:200, Ab8918, Abcam), rabbit anti-CD163 (1:200, Bs-2527R, Bioss), mouse anti-PCNA (1:500, A1111, Santa Cruz), rat anti-BrdU (1:500, OBT003G, Serotec), and rat anti-F4/80 (1:150, MCA497G, BioRad). Secondary antibodies (all diluted 1:1,000): rabbit anti-rat biotinylated (BA-4001, VectorLab), donkey anti-goat Alexa 488 (A11055, Mol Probes), donkey anti-rat Alexa 488 (A21208, Molecular Probes), donkey anti-mouse Alexa 488 (A21202, Life Tech), donkey anti-rabbit Alexa 568 (A10042, Life Tech), donkey anti-goat Alexa 647 (705605147, Jackson IR), donkey anti-rabbit Alexa 647 (A1573, Life Tech), and donkey anti-rat Alexa 647 (712-606-150, Jackson IR). Cell nuclei were stained with 4′,6-diamidino-2-phenylindole dihydrochloride (DAPI) at a concentration of 0.5 μg/μl (Sigma-Aldrich).

### Microscopy and Image Processing

For quantitative analysis of the MBP staining, images were taken with a Zeiss Axioplan (5× objective) and the AxioCam MRC5 Zeiss Imaging Software (version 4.30.01). For the analysis of fluorescent stainings, z-stacks (spanning the whole 40 μm thickness of the section) were generated with a confocal scanning laser microscope (Zeiss LSM 700) equipped with LSM software (ZEN 2012) using the 20× objective and 0.5× zoom. For the figures, representative images were chosen from those taken for analysis. From the confocal images, orthogonal maximum intensity projections were created using the Zen 2012 blue edition software. If necessary, brightness and contrast were adjusted for the whole image.

### Quantitative Analysis of Immunohistochemistry

All image analysis was done blinded (i.e., without knowing group or mouse number) using the software ImageJ 1.48 (GNU General Public License). The percentage area covered by an immunohistological staining was determined using the “Analyze particles” function of ImageJ. For each animal, four tissue sections were used to analyze staining in the two target regions. The light microscopic images of MBP staining were quantified in an area of 160 × 90 μm in the hilus of the dorsal hippocampal DG and an area of 220 × 220 μm in the splenium of the CC. For the fluorescent images of the other markers, first a maximum intensity projection of the z-stacks was created in ImageJ, and then an area of 200 × 100 μm was analyzed in the hilus of the dorsal hippocampal DG and an area of 320 × 320 μm in the splenium of the CC.

For analysis of cell numbers (of Iba-1^+^ PCNA^+^ and Iba-1^+^ BrdU^+^ cells), the total numbers of stained cells within the hilus of dorsal hippocampal DG (without the subgranular zone) and the splenium of the CC were counted in a tenth of a hemisphere using the “Cell Counter” plug-in (cell_counter.jar, version 2, GNU General Public License) for ImageJ and then multiplied per 10.

### Statistical Analysis

Data are shown as means + standard deviation (SD). All statistical analysis was done with Prism 7 (Graphpad Software Inc., San Diego, CA, USA). For the analysis of the statistical significance of the effects of age and treatment on immunohistological parameters, statistical significance was determined with a two-way ANOVA (factors: age and treatment) followed by a Tukey’s *post hoc* test in which each group was compared to all others. The effect of the different treatments on the change of body weight during the experiment were analyzed separately for each age group using a one-way ANOVA with a Tukey’s *post hoc* test or a Kruskal-Wallis one-way analysis followed by a Dunn’s multiple comparisons test depending on whether the data was normally distributed or not (as assessed by a Shapiro-Wilk test). The resulting *p*-values of the comparisons between different treatments of the same age group are represented as: *p < 0.05, **p < 0.01, ***p < 0.001 and ****p < 0.0001, whereas the *p*-values of age-related differences within the same treatment are shown as: ^#^*p* < 0.05, ^##^*p* < 0.01, ^###^*p* < 0.001 and ^####^*p* < 0.0001. Other significant differences (i.e., between different treatments across the two age groups) are not shown.

## Results

### De- and Remyelination in Young and Middle-Aged Mice

**Figure 1:**
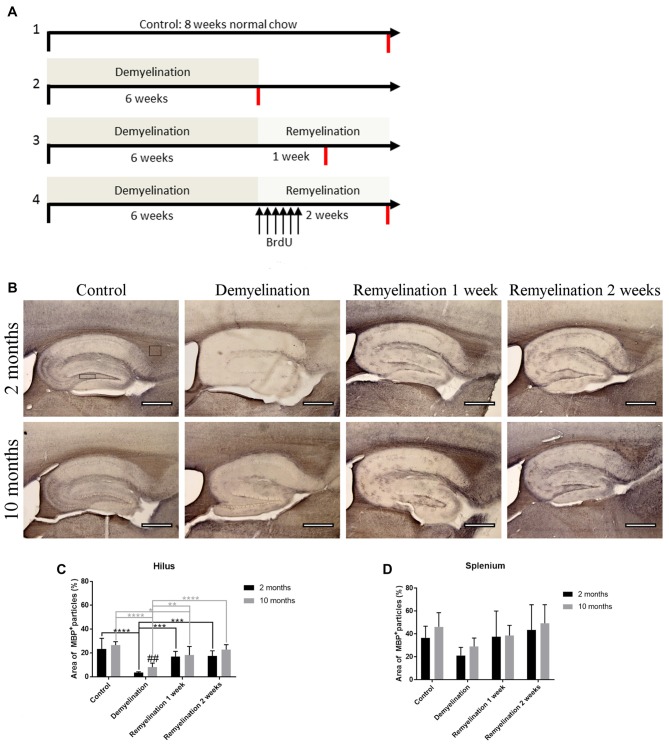
Experimental design and analysis of de- and remyelination. **(A)** Overview of the different treatment groups and the timeline of the experiment. All four treatment groups (controls, demyelination, 1 week remyelination and 2 weeks remyelination) were compared at two different ages: young (starting in 2-month-old animals) and middle-aged (starting in 10-month-old animals). To analyze proliferation and subsequent survival of microglia during remyelination, controls and the remyelination groups received six BrdU injections starting as soon as the demyelination-inducing cuprizone treatment was stopped. The red lines indicate the time of tissue collection. **(B)** Myelination was analyzed using staining for myelin basic protein (MBP). The analyzed regions of interest are indicated by rectangles. **(C)** In the hilus of the dorsal hippocampal dentate gyrus (DG), both age groups were significantly demyelinated, followed by remyelination beginning 1 week after the end of the cuprizone treatment. After six weeks of cuprizone treatment, demyelination was slightly, but significantly, more severe in the young group. **(D)** In the splenium of the corpus callosum (CC), the observed myelin changes during de- and remyelination were less pronounced. Values are shown as means + standard deviation (SD *n* = 6 per group). Statistical significance was evaluated using a two-way ANOVA followed by a Tukey’s *post hoc* test. The *p*-values are indicated in the graphs: treatment effects within the same age group: *p < 0.05, **p < 0.01, ***p < 0.001 and ****p < 0.0001, age-related differences: ^##^*p* < 0.01. Bars: **(B)** 500 μm.

To analyze age-related changes after demyelination, four different groups (control, demyelination, remyelination 1 week, and remyelination 2 weeks) were compared at two different ages. In the young group the 6–8 week treatment started at 2 months, and in the middle-aged group at 10 months of age (for experimental design see Figure 1A). For the analysis of the extent of de- and remyelination, a staining for MBP was used (Figure 1B). In the hilus of the dorsal DG of the hippocampus, both age groups were significantly demyelinated, which was followed by remyelination starting 1 week after cuprizone withdrawal. During demyelination, MBP levels were slightly, but significantly, lower in the younger group (Figure 1C). In the splenium of the CC, regardless of the age group, a reduction of MBP was found in the demyelination group. This observed change in myelination, however, was not significant (Figure 1D). In comparison to the control groups of the same age, no significant differences in the percentage change of body weight over the duration of the experiment were observed in the de- or remyelination groups (Supplementary Figure S1). This indicates that food intake was not negatively affected by the addition of cuprizone to the food pellets.

### Divergent Spatial and Temporal Expression of “General” Microglia Markers

**Figure 2:**
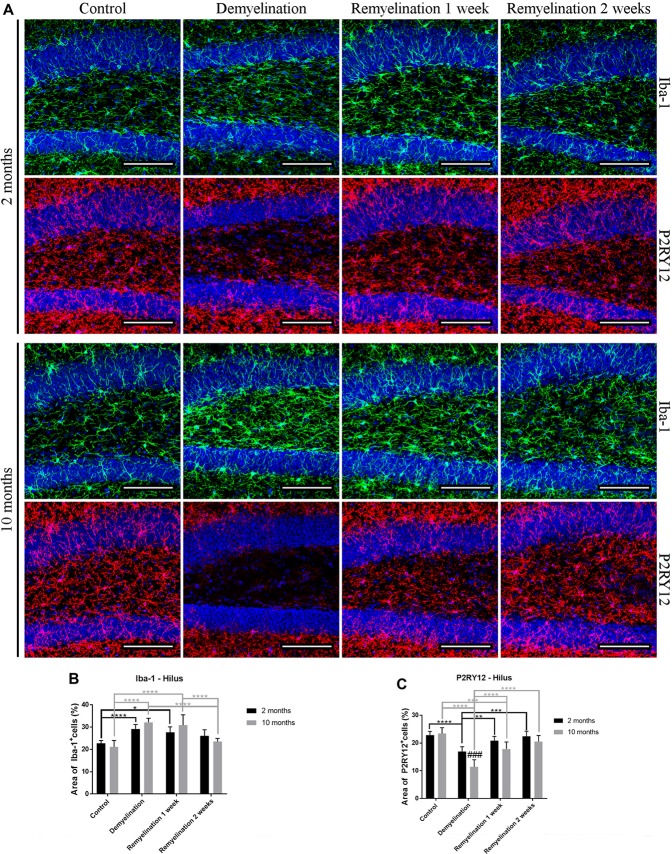
Different patterns of ionized calcium binding adaptor molecule 1 (Iba-1), and P2RY12 during de- and remyelination in the hilus of the DG. **(A)** Iba-1 (green) and P2RY12 (red) labeling. Cell nuclei are stained with 4′,6-diamidino-2-phenylindole dihydrochloride (DAPI; blue). **(B)** In both age groups, Iba-1 staining was significantly increased during demyelination until the first week of remyelination. **(C)** P2RY12 labeling was significantly decreased in both age groups during demyelination, and this was more pronounced and longer lasting in the middle-aged animals. Values are shown as means + SD (*n* = 6 per group). Statistical significance was evaluated using a two-way ANOVA and a Tukey’s *post hoc* test. The *p*-values are indicated in the graphs: treatment effects within the same age group: *p < 0.05, **p < 0.01, ***p < 0.001 and ****p < 0.0001, age-related differences: ^###^*p* < 0.001. Bars: **(A)** 100 μm.

In the hilus of the hippocampal DG (Figure 2A) in both age groups, Iba-1 immunoreactivity was significantly increased during demyelination, which lasted until the first week of remyelination (Figure 2B). P2RY12, however, was significantly down-regulated in both age groups during demyelination (Figure 2C). In the older animals, the down-regulation of P2RY12 was significantly more pronounced than in the younger age group, and persisted for a longer duration (until the first week of remyelination; Figure 2C).

**Figure 3:**
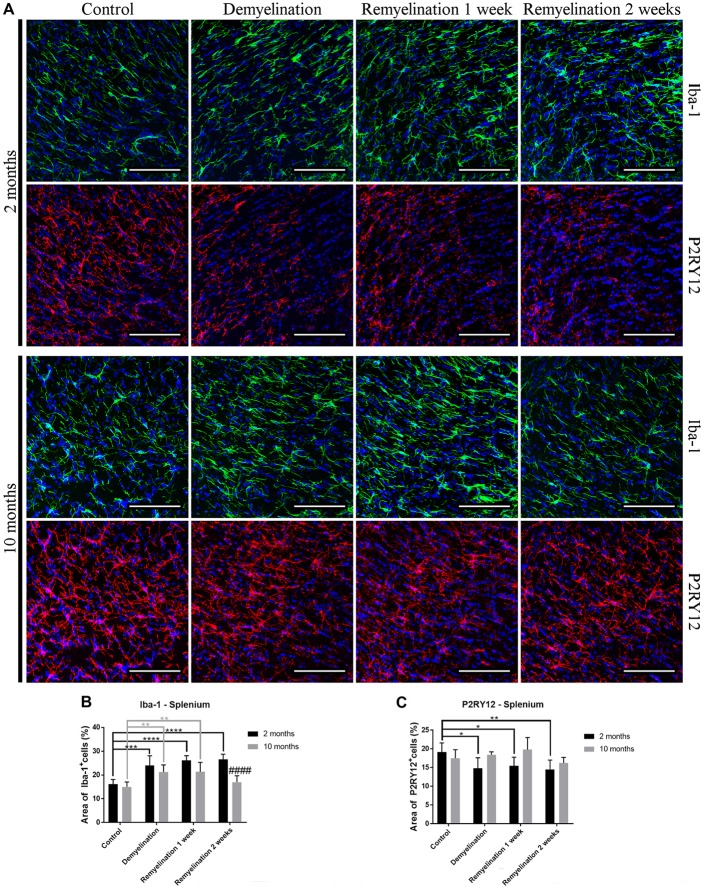
Age- and region-dependent differences in Iba-1 and P2RY12 during de- and remyelination in the splenium of the CC. **(A)** Iba-1 (green) and P2RY12 (red) labeling. Cell nuclei are stained with DAPI (blue). **(B)** In young mice, Iba-1 was up-regulated during all phases of de- and remyelination, while in the middle-aged group Iba-1 returned to control levels after 2 weeks of remyelination, resulting in an age-related difference at this time point. **(C)** A significant reduction of P2RY12 in the splenium was observed only in the younger group during de- and remyelination. In the middle-aged group P2RY12 levels were not changed during de- or remyelination. Values are shown as means + SD (*n* = 6 per group). Statistical significance was evaluated using a two-way ANOVA and a Tukey’s *post hoc* test. The *p*-values are indicated in the graphs: treatment effects within the same age group: *p < 0.05, **p < 0.01, ***p < 0.001 and ****p < 0.0001, age-related differences: ^####^*p* < 0.0001. Bars: **(A)** 100 μm.

In the splenium of the CC (Figure 3A) in the young group, the upregulation of Iba-1 lasted longer than in the hilus of the DG (until the second week of remyelination; Figure 3B). At this time point, the level of Iba-1 expression in the splenium of the older group had already returned to control levels resulting in a significant age-related difference in Iba-1 expression (Figure 3B). Interestingly, a significant downregulation of P2RY12 in the splenium was observed only in the younger group during de- and remyelination (Figure 3C). In the older group, P2RY12 expression did not change significantly in the splenium in response to de- or remyelination (Figure 3C).

**Figure 4:**
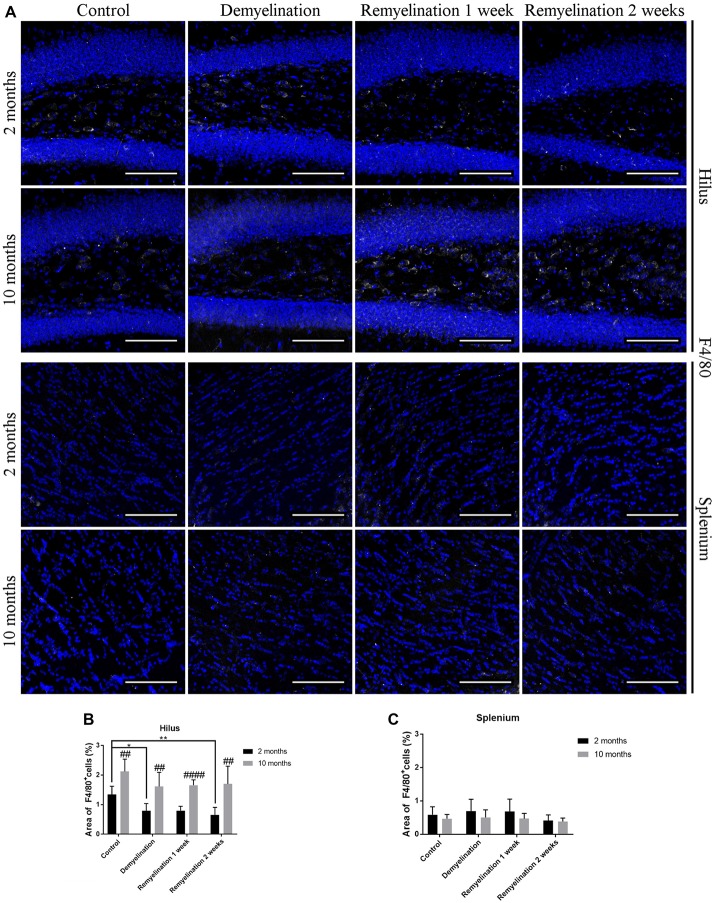
F4/80 levels are affected by age and cuprizone treatment in the hilus of the DG, but not in the splenium of the CC. **(A)** F4/80 (white) staining in the hilus of the DG (two upper rows) and the splenium of the CC (two lower rows). Cell nuclei are stained with DAPI (blue). F4/80 is relatively low in all groups. **(B)** In the hilus of the hippocampal DG, F4/80 levels were higher in middle-aged groups compared to young ones. In young mice, F4/80 was significantly reduced after demyelination and 2 weeks of remyelination. **(C)** In contrast, in the splenium of the CC no significant changes in F4/80 expression were found. Values are shown as means + SD (*n* = 6 per group). Statistical significance was evaluated using a two-way ANOVA followed by a Tukey’s *post hoc* test. The *p*-values are indicated in the graphs: treatment effects within the same age group: *p < 0.05 and **p < 0.01, age-related differences: ^##^*p* < 0.01 and ^####^*p* < 0.0001. Bars: **(A)** 100 μm.

In the hilus of the hippocampal DG (Figure 4A, upper rows), we found a significantly higher F4/80 expression in all older groups compared to the young ones (Figure 4B). In addition, F4/80 was reduced in all young groups following de- and remyelination (significant after demyelination and 2 weeks of remyelination; Figure 4B). In contrast, in the splenium of the CC (Figure 4A, lower rows), no significant changes in F4/80 expression were observed (Figure 4C).

### CD68 and MHCII React Differently to De- and Remyelination

**Figure 5:**
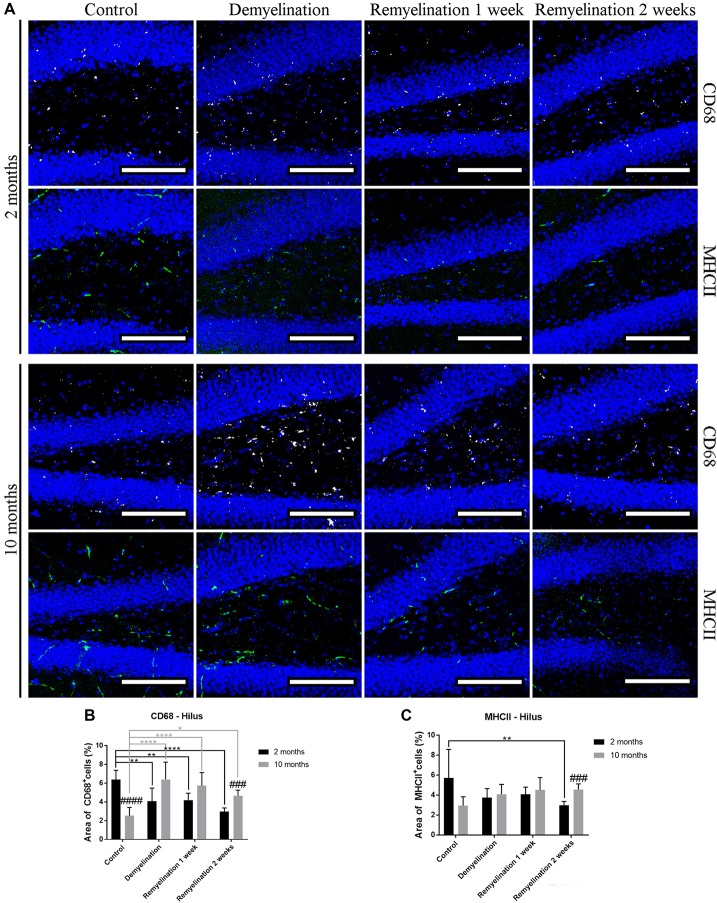
Age-related heterogeneity in CD68 and major histocompatibility complex II (MHCII) in the hilus of the hippocampal DG. **(A)** CD68 (white) and MHCII (green) labeling. Cell nuclei are stained with DAPI (blue). **(B)** There was an age-related difference of CD68 staining in the control groups (higher in the younger controls). In the young groups, the percentage of CD68 staining decreased significantly during de- and remyelination, whereas the opposite was observed for the middle-aged groups. These different patterns led to a significantly higher CD68 level in the middle-aged group in comparison to the younger one after 2 weeks of remyelination. **(C)** MHCII expression was significantly reduced in the younger group after 2 weeks of remyelination. At the same time point, there was a significant age-related difference, with higher levels of MHCII in the middle-aged group. Values are shown as means + SD (*n* = 6 per group). Statistical significance was evaluated using a two-way ANOVA and a Tukey’s *post hoc* test. The *p*-values are indicated in the graphs: treatment effects within the same age group: *p < 0.05, **p < 0.01 and ****p < 0.0001, age-related differences: ^###^*p* < 0.001 and ^####^*p* < 0.0001. Bars: **(A)** 100 μm.

**Figure 6:**
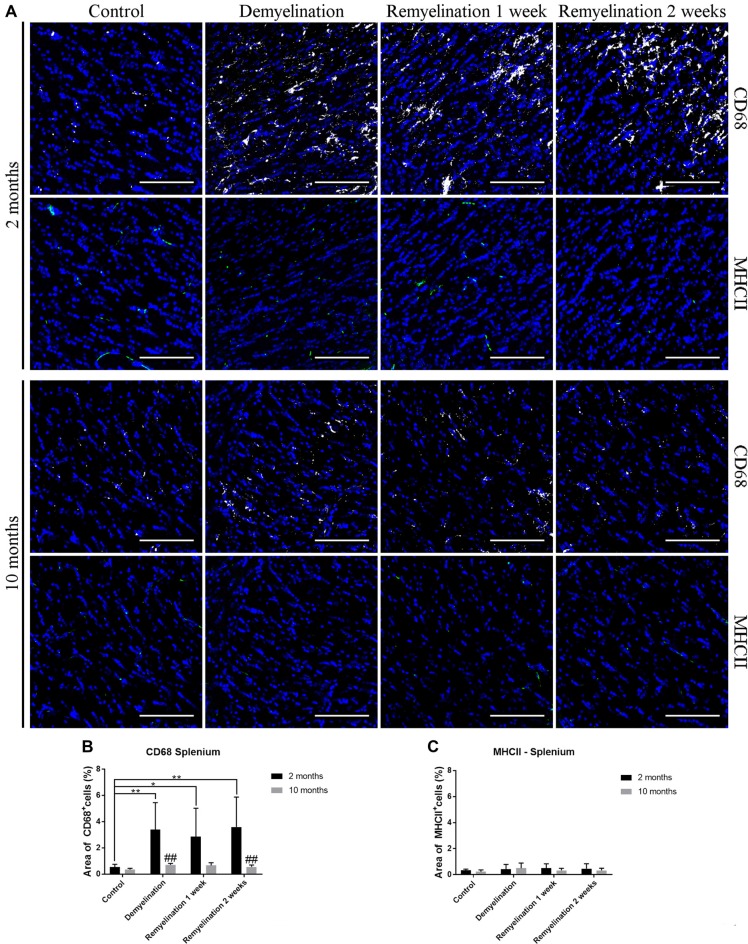
Age-related differences in CD68, but not in MHCII, in splenium of the CC. **(A)** CD68 (white) and MHCII (green) labeling. Cell nuclei are stained with DAPI (blue). **(B)** CD68 was significantly increased during de- and remyelination only in the younger group leading to significant age-related differences (with higher values in the younger group) after demyelination and 2 weeks of remyelination. **(C)** MHCII staining was not significantly changed by de- or remyelination in both age groups. Values are shown as means + SD (*n* = 6 per group). Statistical significance was evaluated using a two-way ANOVA followed by a Tukey’s *post hoc* test. The *p*-values are indicated in the graphs: treatment effects within the same age group: *p < 0.05 and **p < 0.01, age-related differences: ^##^*p* < 0.01. Bars: **(A)** 100 μm.

When analyzing CD68 expression in the hilus of the hippocampal DG (Figure 5A), the first unexpected finding was that the expression of this marker was higher in the younger controls compared to the middle-aged ones (Figure 5B). In the younger de- and remyelination groups, the percentage of CD68 staining decreased significantly in comparison to controls, whereas the opposite was observed for the older groups (Figure 5B). These opposed regulation patterns resulted in a significantly higher CD68 expression in the middle-aged group after 2 weeks of remyelination in comparison to the younger one (Figure 5B). The percentage of MHCII staining was subtly, but significantly, reduced in the hippocampal hilus of the younger group after 2 weeks of remyelination (Figure 6C). In contrast, in the middle-aged group, a small, not significant, increase in MHCII was observed during de- and remyelination, which resulted in a significant age-related difference after 2 weeks of remyelination with higher levels of MHCII in the middle-aged group (Figure 5C).

In the splenium of the CC (Figure 6A), there were no differences between the controls of the two age groups, neither in the expression of CD68 (Figure 6B), nor MHCII (Figure 6C). Interestingly, de- and remyelination significantly increased CD68 only in the younger group resulting in significant age-related differences after demyelination and 2 weeks of remyelination (Figure 5C). MHCII expression was not significantly altered during de- or remyelination in both age groups in the splenium, and the expression of this marker was generally much lower than in the DG hilus (Figure 6C).

### TSPO, but Not Marco, Is Influenced by De- and Remyelination

**Figure 7:**
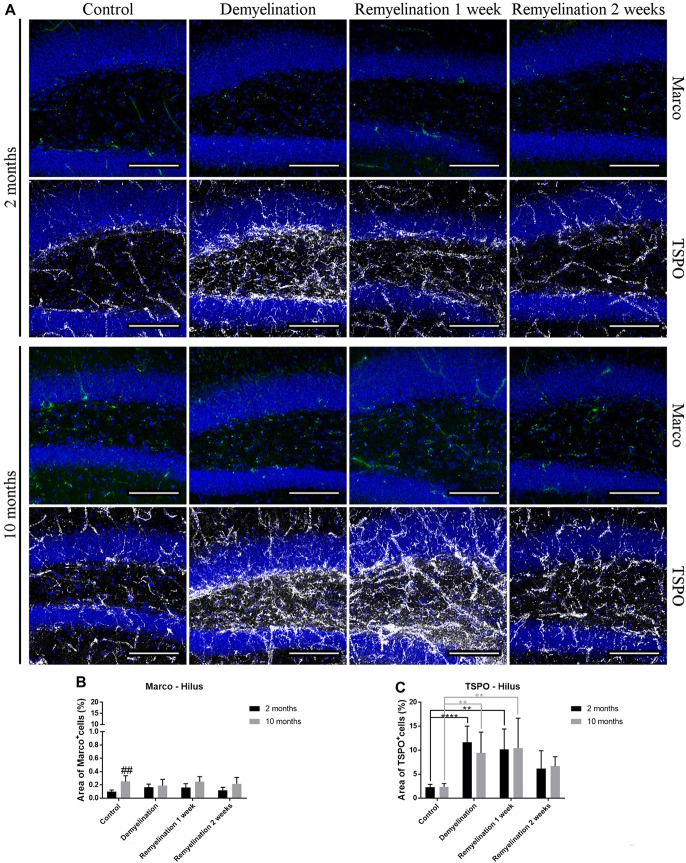
Translocator protein 18 kD (TSPO), but not macrophage receptor with collagenous structure (Marco), reacts to de- and remyelination in the hilus of the hippocampal DG. **(A)** Marco (green) and TSPO (white) labeling. Cell nuclei are stained with DAPI (blue). **(B)** Marco staining was significantly higher in the middle-aged control group compared to the young one. De- and remyelination did not significantly change the levels of Marco. **(C)** TSPO was significantly increased during demyelination and the first week of remyelination in both age groups. Values are shown as means + SD (*n* = 6 per group). Statistical significance was evaluated using a two-way ANOVA followed by a Tukey’s *post hoc* test **(C)**. The *p*-values are indicated in the graphs: treatment effects within the same age group: **p < 0.01 and ****p < 0.0001, age-related differences: ^##^*p* < 0.01. Bars: **(A)** 100 μm.

In the hilus of the hippocampal DG (Figure 7A), a significantly higher expression of Marco was found in the older control group compared to the younger one (Figure 7B). De- and remyelination, however, did not significantly affect the (very low) expression of this classical pro-inflammatory marker. In the same region, there were no age-related differences in TSPO expression, but in both age groups a significant TSPO increase was found during demyelination and the first week of remyelination (Figure 7C).

**Figure 8:**
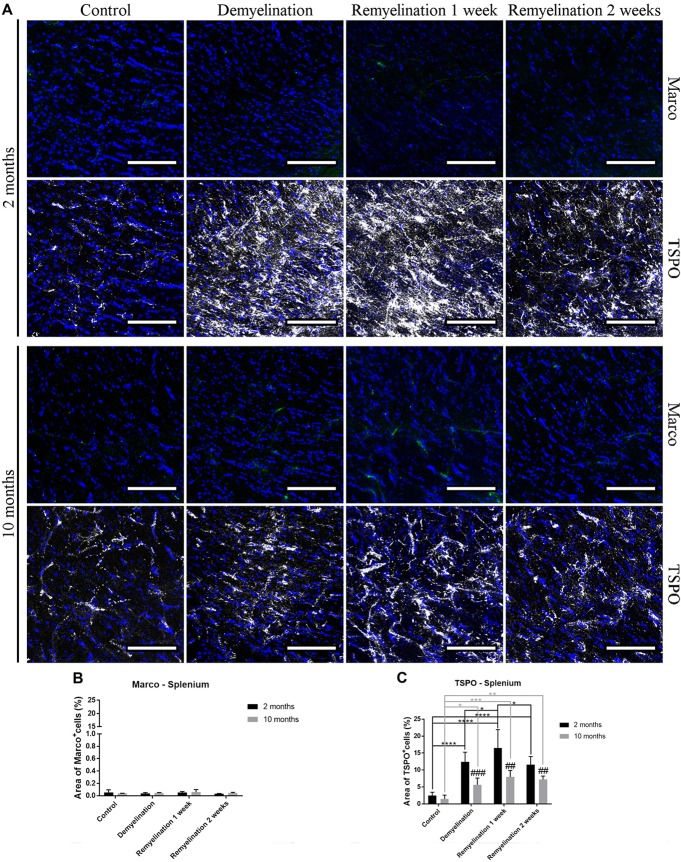
Age-related differences in cuprizone-induced increase of TSPO in the splenium of the CC, while Marco remains unaffected. **(A)** Marco (green) and TSPO (white) labeling. Cell nuclei are stained with DAPI (blue). **(B)** No significant effects of age or treatment on Marco levels were observed. **(C)** In both age groups, TSPO was significantly increased during de- and remyelination. TSPO levels were significantly higher in the younger de- and remyelination groups. Values are shown as means + SD (*n* = 6 per group). Statistical significance was evaluated using a two-way ANOVA followed by a Tukey’s *post hoc* test **(C)**. The *p*-values are indicated in the graphs: treatment effects within the same age group: *p < 0.05, **p < 0.01, ***p < 0.001 and ****p < 0.0001, age-related differences: ^##^*p* < 0.01 and ^###^*p* < 0.001. Bars: **(A)** 100 μm.

In the splenium of the CC (Figure 8A), no significant effects of age or treatment on Marco expression were found (Figure 8B). In contrast, TSPO was significantly increased in both age groups during de- and remyelination, however, the TSPO levels were significantly higher in the younger groups during de- and remyelination (Figure 8C).

### Age and Region Affect Induction of CD206 and CD163 During Remyelination

**Figure 9:**
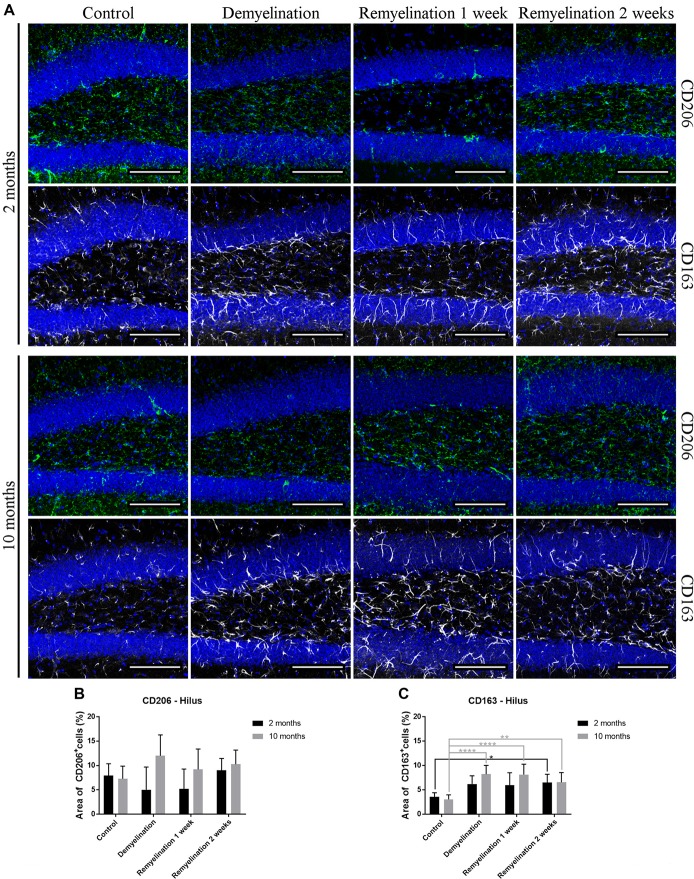
Age-related differences in cuprizone-induced increase of CD163 in the hilus of the hippocampal DG, while CD206 is unchanged. **(A)** CD206 (green) and CD163 (white) labeling. Cell nuclei are stained with DAPI (blue). **(B)** In both age groups, de- and remyelination did not significantly affect CD206 staining. **(C)** In the middle-aged group, CD163 was significantly upregulated during de- and remyelination. In the younger group, the CD163 increase was only significant 2 weeks after remyelination. Values are shown as means + SD (*n* = 6 per group). Statistical significance was evaluated using a two-way ANOVA followed by a Tukey’s *post hoc* test **(C)**. The *p*-values are indicated in the graphs: treatment effects within the same age group: *p < 0.05, **p < 0.01 and ****p < 0.0001. Bars: **(A)** 100 μm.

**Figure 10:**
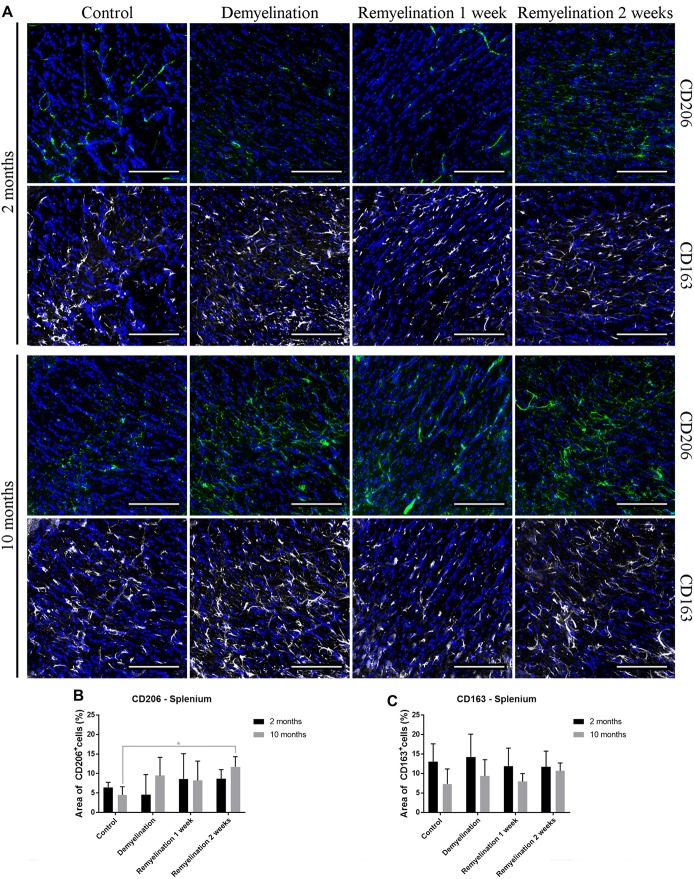
Increase of CD206 during remyelination in middle-aged mice in the splenium of the CC, while CD163 is unaffected. **(A)** CD206 (green) and CD163 (white) labeling. Cell nuclei are stained with DAPI (blue). **(B)** CD206 was significantly elevated in the middle-aged group after 2 weeks of remyelination. **(C)** CD163 was not significantly affected by age or treatment. Values are shown as means + SD (*n* = 6 per group). Statistical significance was evaluated using a two-way ANOVA followed by a Tukey’s *post hoc* test **(C)**. The *p*-values are indicated in the graphs: treatment effects within the same age group: *p < 0.05. Bars: **(A)** 100 μm.

In the hippocampal hilus (Figure 9A), de- and remyelination had no significant effect on the expression of CD206 in either age group (Figure 9B). In the middle-aged group, there was a significant upregulation of CD163 during the whole de- and remyelination phase, while in the younger group a similar pattern was observed, but the increase in CD163 was only significant 2 weeks after remyelination (Figure 9C). In the splenium (Figure 10A), CD206 was only significantly elevated in the middle-aged group after 2 weeks of remyelination (Figure 10B). For CD163, no significant changes were observed in this region (Figure 10C).

### Increased Proliferation of Microglia in the Splenium of the CC in the Young Group

**Figure 11:**
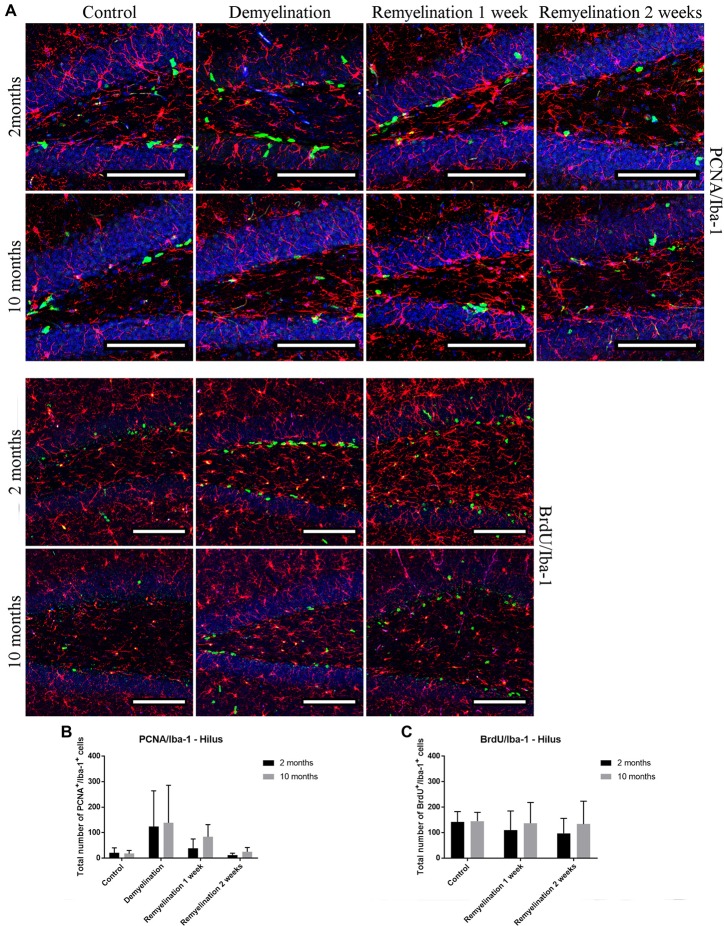
No significant changes in numbers of proliferating microglia (PCNA^+^ and BrdU^+^) in the hilus of the hippocampal DG. **(A)** Proliferating cell nuclear antigen (PCNA; green) and Iba-1 (red) labeling. Cell nuclei are stained with DAPI (blue). **(B)** Bromodeoxyuridine (BrdU; green) and Iba-1 (red) labeling. Cell nuclei are stained with DAPI (blue). **(C)** No significant effects on the number of proliferating PCNA^+^ microglia was observed. **(D)** The number of Iba-1^+^ BrdU^+^ cells was also not affected during de- or remyelination in either age group. Values are shown as means + SD (*n* = 6 per group). Statistical significance was evaluated using a two-way ANOVA and a Tukey’s *post hoc* test. Bars: **(A)** 100 μm.

No significant increase in proliferating microglia (PCNA^+^) was found during de- or remyelination in the hippocampal hilus (Figure 11A), which could be due to the very high variance within the demyelination groups in both age groups (Figure 11C). In accordance with these results, the number of Iba-1^+^ BrdU^+^ cells (Figure 11B) was also not increased during de- or remyelination in either age group (Figure 11D).

**Figure 12:**
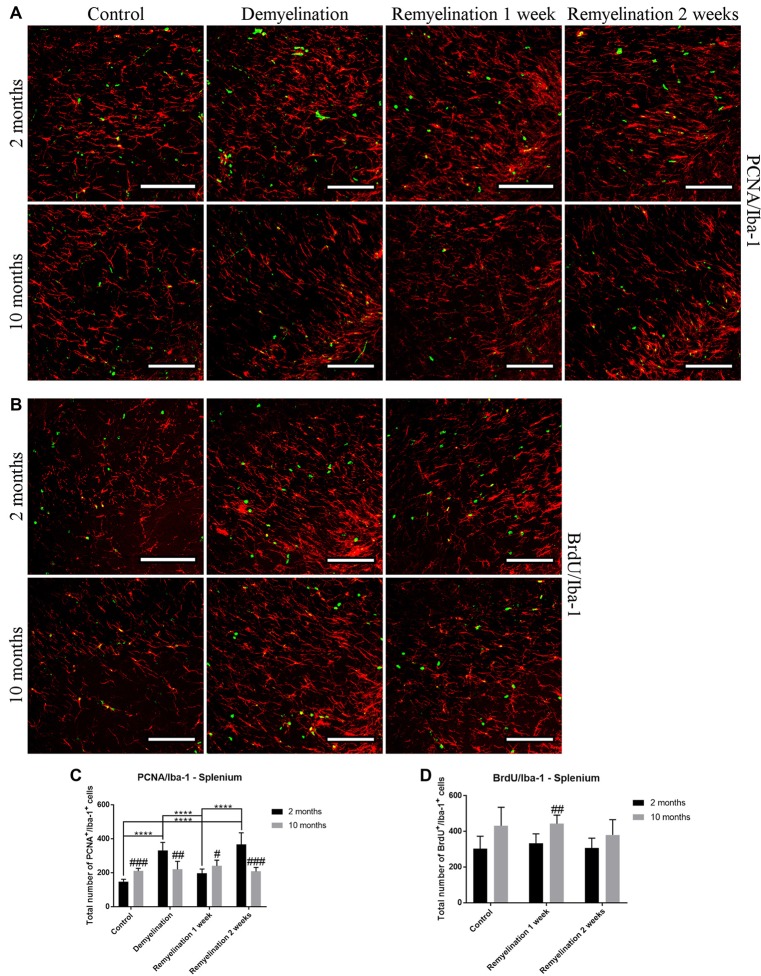
Age-related differences in the number of proliferating microglia (PCNA^+^ and BrdU^+^) in the splenium of the CC. **(A)** PCNA (green) and Iba-1 (red) labeling. **(B)** BrdU (green) and Iba-1 (red) staining. **(C)** The number of proliferating PCNA^+^ microglia was significantly increased in the middle-aged control group compared to the younger one. In young mice, there was a significant increase in PCNA^+^ microglia after demyelination and 2 weeks of remyelination. Thus, age-related differences in PCNA^+^ Iba-1^+^ cell numbers were found in all treatment groups. **(D)** The number of BrdU^+^ microglia was higher in the middle-aged group after 1 week of remyelination (a similar tendency was also observed in the other two groups). De- or remyelination did not affect the number of BrdU^+^ Iba-1^+^ microglia. Values are shown as means + SD (*n* = 6 per group). Statistical significance was evaluated using a two-way ANOVA and a Tukey’s *post hoc* test. The *p*-values are indicated in the graphs: treatment effects within the same age group: ****p < 0.0001, age-related differences: ^#^*p* < 0.05, ^##^*p* < 0.01 and ^###^*p* < 0.001. Bars: **(A)** 100 μm.

In the splenium of the CC (Figure 12A), the number of proliferating PCNA^+^ microglia was significantly higher in the middle-aged controls than in the young ones. However, only the younger group was affected by the cuprizone treatment: in this age group, there was a significant increase in proliferating microglia after demyelination and the second week of remyelination. This dynamic resulted in age-related differences at all time points (Figure 12C). The number of BrdU^+^ microglia in the splenium (Figure 12B) was higher in all middle-aged groups in comparison to the younger ones, however, this was only significant after 1 week of remyelination (Figure 12D). This increase of Iba-1^+^ BrdU^+^ cells fits together with the higher proliferation in the middle-aged groups that was observed in the PCNA staining in the controls and 1 week after remyelination during which BrdU was administered to the remyelination groups (Figure 12C). However, de- or remyelination did not affect the number of BrdU^+^ Iba-1^+^ microglia in either age group (Figure 12D). This is in accordance with the PCNA data, since in both age groups the number of proliferating PCNA^+^ microglia was not increased after 1 week of remyelination (during which BrdU was injected).

## Discussion

The results of the present study show that microglia in the middle-aged CNS are already subtly changed in comparison to young microglia and that they also react differently to demyelination in the cuprizone model. The observed age-related microglial changes differed between the two analyzed regions, hilus of the dorsal hippocampal DG a gray matter region functionally involved in learning and memory, and the splenium of the CC as a typical white matter region.

In both age groups, the analysis of MBP confirmed cuprizone-induced de- and remyelination, which was even more pronounced in the analyzed hippocampal region than the splenium of the CC. This is in agreement with work by others showing that in the cuprizone model demyelination occurs in the splenium of the CC (Steelman et al., 2012; Le Blon et al., 2016; Tagge et al., 2016) and the hippocampus (Hoffmann et al., 2008; Koutsoudaki et al., 2009; Norkute et al., 2009; Sun et al., 2016). Thus, the cuprizone model is well suited for studies on demyelination in the hippocampus, a region which is also affected in human MS patients (Geurts et al., 2007; Sicotte et al., 2008; Dutta et al., 2011; Hulst et al., 2015; Planche et al., 2017) with the hippocampal DG being particularly vulnerable at earliest stages of MS progression (Planche et al., 2018).

For analyzing differences between young and middle-aged mice during de- and remyelination, we first used Iba-1, a classical microglia marker, since it is highly expressed in microglia and is increased after pro-inflammatory stimulation (e.g., Mori et al., 2000; Ito et al., 2001). In the analyzed gray matter region, Iba-1 showed a similar pattern of increase during demyelination and the first week of remyelination for both age groups. However, in the splenium of the CC, the younger group showed a prolonged activation. This pattern fits to the observed changes in TSPO, a pro-inflammatory marker, which, in contrast to Iba-1, is not only expressed in microglia/macrophages, but also in astrocytes. For both Iba-1 and TSPO, a higher induction was observed in the younger group after cuprizone treatment in the analyzed white matter region, whereas there were no age-related differences in the gray matter region. TSPO is of particular interest, since PET tracer studies targeting TSPO have found convincing evidence that microglial activation indeed correlates with MS symptoms: in MS patients, this marker is not only increased in MS lesions and normal appearing gray and white matter, but also correlates with age, disease progression and disability (reviewed in Airas et al., 2018).

An unexpected finding was the significantly lower expression of CD68 in the hilus of the DG in the older controls, since it is well described that CD68 increases during aging in different brain regions (reviewed in Mosher and Wyss-Coray, 2014; Norden et al., 2015). However, one study, which performed a detailed analysis of microglia in different brain regions, found no significant increase in CD68 in old mice (20-months-old) in the DG under control conditions or after LPS injection (Hart et al., 2012). During de- and remyelination, CD68 expression tended to be higher in middle-aged mice than in the younger groups in the hilus of the DG, while the opposite was observed in the splenium of the CC, in which CD68 was up-regulated to much higher levels in the younger groups. Similar to the Iba-1 and TSPO results, this indicates a more extensive and longer lasting activation of microglia in the splenium of younger mice. However, at this point, we do not know how myelin repair is affected by the higher levels of activation markers in young microglia. It could be speculated, for example, that this age-related difference may be indicative for a more active phagocytosis in younger microglia, which has been found in a focal demyelination model (Rawji et al., 2018).

As expected, we found only low levels of MHCII in microglia under control conditions. However, the staining pattern of this marker seems to be associated at least in part with blood vessels, which could be explained by the staining of vessel-associated antigen presenting perivascular macrophages or dendritic cells. Moreover, brain pericytes are also able to express MHCII and inflammatory stimuli (such as IFN-γ) increase the MHCII expression in this perivascular cell type (Pieper et al., 2014). Surprisingly, a reduction of MHCII was found in the young group after 2 weeks of remyelination in the hippocampal DG. This, however, may be of limited functional relevance, since the observed MHCII levels are very low, and in particular are highly variable in the young control.

For two other markers that are induced by pro-inflammatory stimuli, F4/80 and Marco, the expected increase in expression was observed in middle-aged mice, but only in the analyzed gray matter region. Here, Marco was significantly higher in the older controls, and F4/80 was significantly up-regulated in all middle-aged groups, while surprisingly the F4/80 expression in the young groups was further reduced during de- and remyelination. Since most studies on microglial aging are done in old, not middle-aged mice, it is difficult to find comparable results in the literature. An age-related increase in F4/80 was found in the hippocampus of old mice (24-months-old) in one study (Boehme et al., 2014), whereas another group found that F4/80 increased in white matter regions, but not in the DG of old mice (20-months-old; Hart et al., 2012). While an injection of an M1-inducing cytokine cocktail increased Marco expression in young (6-month-old), middle-aged (12-month-old) and old mice (24-month-old mice), that study, however, reported no differences between vehicle-treated animals of different ages (Lee et al., 2013).

P2RY12 is expressed in microglia under physiological conditions, and it has been shown that it can also be used to distinguish microglia from infiltrating macrophages in mice, which is not possible with other commonly used microglia markers (Butovsky et al., 2014). Additionally, P2RY12 is being discussed as an M2-like anti-inflammatory marker, since it has been shown *in vitro* that P2RY12 expression is increased in microglia following anti-inflammatory stimuli (Moore et al., 2015; Beaino et al., 2017). In the present study, a significant reduction of P2RY12 was found in both age groups in the hippocampal DG, which was even more severe in the middle-aged group. In the splenium, a similar pattern was only observed in the younger group. This reduction in P2RY12 coincided with an upregulation in Iba-1 and TSPO (which were both less pronounced in the splenium of the middle-aged group), suggesting that these markers are inversely regulated. These results are in accordance with other studies, which observed a reduced expression of P2RY12 in active inflammatory lesions in MS patients (Beaino et al., 2017; Mildner et al., 2017; Zrzavy et al., 2017). However, in a study investigating experimental autoimmune encephalomyelitis (EAE), an MS model in which pathology is driven mainly by CD4^+^ T cells (reviewed in Sonar and Lal, 2017), an increased P2RY12 expression was found during the remission phase (Beaino et al., 2017), which indicates that the regulation of P2RY12 may be more complex. Zrzavy et al. (2017) analyzed P2RY12 and the anti-inflammatory microglia markers CD206 and CD163 in parallel in MS patients. This study showed a different pattern for these markers: P2RY12 was lost in active lesions but reappeared in the center of inactive lesions while the expression of CD163 and CD206 were highest in late active/inactive lesions (Zrzavy et al., 2017). In the present study, we also did not find a correlation between the changes in P2RY12 and the other analyzed M2-like markers, but rather it seems that P2RY12 is negatively correlated with pro-inflammatory markers.

Microglia have been shown to change their activation state during phagocytosis of myelin to more anti-inflammatory and pro-regenerative traits (Boven et al., 2006; van Rossum et al., 2008; Neumann et al., 2009). The anti-inflammatory marker CD206 (which is not only found in microglia, but also in astrocytes) was only significantly up-regulated in the analyzed white matter region after 2 weeks of remyelination in the middle-aged group. However, the CD206 expression was highly variable, particularly during demyelination and early remyelination. In contrast, CD163, a marker for microglia and perivascular macrophages, was significantly increased in both age groups, but this process started earlier in the middle-aged group and was only observed in the analyzed gray matter region. Thus, it seems that middle-aged mice are more likely to display M2-like characteristics than young ones. This difference may have consequences for remyelination, since in MS patients, the M2 marker CD206 is only increased in lesions with ongoing myelin repair (Butovsky et al., 2006; Miron et al., 2013). Others also have found an age-related increase in CD206 in microglia isolated from the cortex of old (24-month-old) mice (Zöller et al., 2018). In contrast, in a focal demyelination model using parabiotic pairs of aged mice (10–12 months-old), it has been shown that there is a lower number of M2-polarized microglia/macrophages (identified with the mannose receptor CD206) in demyelinated lesion, while at the same time remyelination efficiency is reduced. A parabiotic pairing of an aged mouse with a younger one (5–6 weeks-old) increased both the number of lesion-associated M2-polarized cells and remyelination in the older parabiotic partner (Miron et al., 2013). The contrasting results from Miron and colleagues and our study possibly could be due to differences in the investigated demyelination models—e.g., the longer de- and remyelination phases in the cuprizone model. Additionally, at the two analyzed time points, we did not find a difference in the extent of remyelination between the two age groups. However, it would be interesting to do functional studies with M2-polarized microglia/macrophages from aged mice after cuprizone-induced demyelination to find out whether they are indeed beneficial for myelin repair.

A significant increase of PCNA^+^ proliferating microglia was only observed in the splenium of the CC of the younger group after demyelination and the second week of remyelination. A possible explanation for the few changes in proliferation could be that at 6 weeks of demyelination, which was analyzed in the present study, the peak of microglial proliferation that occurs at 4.5 weeks had already passed (Matsushima and Morell, 2001; Praet et al., 2014). The slightly higher number of Iba-1^+^ PCNA^+^ and Iba-1^+^ BrdU^+^ cells in the splenium of middle-aged healthy controls in comparison to young ones was unexpected, since it was shown that microglia have a relatively stable turnover rate during aging in different brain regions, such as the CC (Askew et al., 2017). However, in a stroke model an increased number of Iba-1^+^ BrdU^+^ microglia has been found in middle-aged mice (13–14 months; Moraga et al., 2015).

Traditionally, microglial activation has often been grouped into the classical pro-inflammatory or “M1” activation and the alternative, more anti-inflammatory, “M2” activation with functions in wound healing and regulation of immune responses (Colton, 2009; Olah et al., 2012; Boche et al., 2013). This classification has been derived originally from peripheral macrophages, however its usefulness has been increasingly challenged (Martinez and Gordon, 2014), in particular for the analysis of microglia (Ransohoff, 2016) as it is probably an oversimplification. Nevertheless, the markers which have been described as more M1- or M2-like are still popular targets for microglia analyses. Our results clearly show that immunohistochemical markers for microglia are useful, but great caution has to be taken when it comes to the interpretation of markers as being representative for specific microglia activity states such as M1, M2, pro- or anti-inflammatory. The markers which are currently used rather highlight the presence of a certain cell component, which, in turn, may more or less correlate with a certain microglia function. Along these lines, the present data clearly shows that the expression patterns of markers that are considered to be within the same “activation category,” i.e., either pro- or anti-inflammatory, differ strongly.

The present study examines microglial alterations only after a relatively acute demyelination period. For follow-up studies, it would be interesting to examine age-related differences in microglial activation at different ages and after repeated and/or longer demyelination periods with the ultimate goal to better model the situation in MS patients suffering from cycles of relapse and remission or from chronic progressive pathology. Also, it would also be important to study both sexes in parallel—the present study only analyzed male mice.

Our results show that microglia changes already start during middle-age, but that they need to be carefully analyzed, in particular, because there are also region-dependent differences. In healthy controls, age-related microglial changes were more pronounced in the analyzed gray matter region (resulting in more F4/80 and Marco as well as less CD68 in middle-aged mice). However, after cuprizone-induced demyelination, specifically in the analyzed white matter region, the activation of microglia in the young CNS seems to be stronger and longer lasting than in the middle-aged CNS (as indicated by increased Iba-1 and TSPO as well as reduced P2RY12). Interestingly, anti-inflammatory characteristics (increased CD206 and CD163) tend to be more pronounced in middle-aged mice. How these age-related microglial alterations might affect regeneration and myelin repair after prolonged or recurrent demyelination requires further investigation.

## Data Availability

The datasets used and/or analyzed during the current study are available from the corresponding author on reasonable request.

## Author Contributions

BK designed the study, collected, analyzed and interpreted data and drafted the manuscript. HM collected, analyzed and interpreted data. SL and CB helped with data collection. FR participated in the discussion of the results. LA participated in the study design and coordination, and the discussion of the results. All authors contributed to revising the manuscript and approved the final version.

## Conflict of Interest Statement

The authors declare that the research was conducted in the absence of any commercial or financial relationships that could be construed as a potential conflict of interest.

## Supplementary Material

FIGURE S1 Weight changes during the experiment. Percentage weight changes between the first and the last 2 days of the experiment in **(A)** young and **(B)** middle-aged mice. In both age groups, no significant differences were observed in relative weight changes in comparison to the respective controls. Values are shown as means + SD (*n* = 6 per group). Statistical significance was evaluated using a one-way ANOVA and a Tukey’s *post hoc* test **(A)** and a Kruskal-Wallis one-way analysis followed by a Dunn’s multiple comparisons test **(B)**.

## References

[B1] Airas L. Nylund M. Rissanen E. (2018). Evaluation of microglial activation in multiple sclerosis patients using positron emission tomography. Front. Neurol. 9:181. 10.3389/fneur.2018.00181 29632509 PMC5879102

[B2] Askew K. Li K. Olmos-Alonso A. Garcia-Moreno F. Liang Y. Richardson P.. (2017). Coupled proliferation and apoptosis maintain the rapid turnover of microglia in the adult brain. Cell Rep. 18, 391–405. 10.1016/j.celrep.2016.12.041 28076784 PMC5263237

[B3] Baecher-Allan C. Kaskow B. J. Weiner H. L. (2018). Multiple sclerosis: mechanisms and immunotherapy. Neuron 97, 742–768. 10.1016/j.neuron.2018.01.021 29470968

[B4] Bardou I. Brothers H. M. Kaercher R. M. Hopp S. C. Wenk G. L. (2013). Differential effects of duration and age on the consequences of neuroinflammation in the hippocampus. Neurobiol. Aging 34, 2293–2301. 10.1016/j.neurobiolaging.2013.03.034 23639208 PMC3706469

[B5] Beaino W. Janssen B. Kooij G. van der Pol S. M. A. van Het Hof B. van Horssen J.. (2017). Purinergic receptors P2Y12R and P2X7R: potential targets for PET imaging of microglia phenotypes in multiple sclerosis. J. Neuroinflammation 14:259. 10.1186/s12974-017-1034-z 29273052 PMC5741931

[B6] Bennett M. L. Bennett F. C. Liddelow S. A. Ajami B. Zamanian J. L. Fernhoff N. B.. (2016). New tools for studying microglia in the mouse and human CNS. Proc. Natl. Acad. Sci. U S A 113, E1738–E1746. 10.1073/pnas.1525528113 26884166 PMC4812770

[B7] Boche D. Perry V. H. Nicoll J. A. (2013). Review: activation patterns of microglia and their identification in the human brain. Neuropathol. Appl. Neurobiol. 39, 3–18. 10.1111/nan.12011 23252647

[B8] Boehme M. Guenther M. Stahr A. Liebmann M. Jaenisch N. Witte O. W.. (2014). Impact of indomethacin on neuroinflammation and hippocampal neurogenesis in aged mice. Neurosci. Lett. 572, 7–12. 10.1016/j.neulet.2014.04.043 24796813

[B9] Boven L. A. Van Meurs M. Van Zwam M. Wierenga-Wolf A. Hintzen R. Q. Boot R. G.. (2006). Myelin-laden macrophages are anti-inflammatory, consistent with foam cells in multiple sclerosis. Brain 129, 517–526. 10.1093/brain/awh707 16364958

[B10] Brandenburg L. O. Konrad M. Wruck C. J. Koch T. Lucius R. Pufe T. (2010). Functional and physical interactions between formyl-peptide-receptors and scavenger receptor MARCO and their involvement in amyloid beta 1–42-induced signal transduction in glial cells. J. Neurochem. 113, 749–760. 10.1111/j.1471-4159.2010.06637.x 20141570

[B11] Braun B. J. Slowik A. Leib S. L. Lucius R. Varoga D. Wruck C. J.. (2011). The formyl peptide receptor like-1 and scavenger receptor MARCO are involved in glial cell activation in bacterial meningitis. J. Neuroinflammation 8:11. 10.1186/1742-2094-8-11 21299846 PMC3040686

[B12] Browne P. Chandraratna D. Angood C. Tremlett H. Baker C. Taylor B. V.. (2014). Atlas of multiple sclerosis 2013: a growing global problem with widespread inequity. Neurology 83, 1022–1024. 10.1212/wnl.0000000000000768 25200713 PMC4162299

[B13] Burudi E. M. E. Riese S. Stahl P. D. Regnier-Vigouroux A. (1999). Identification and functional characterization of the mannose receptor in astrocytes. Glia 25, 44–55. 10.1002/(sici)1098-1136(19990101)25:1<44::aid-glia5>3.0.co;2-c 9888297

[B14] Butovsky O. Jedrychowski M. P. Moore C. S. Cialic R. Lanser A. J. Gabriely G.. (2014). Identification of a unique TGF-β-dependent molecular and functional signature in microglia. Nat. Neurosci. 17, 131–143. 10.1038/nn.3599 24316888 PMC4066672

[B15] Butovsky O. Landa G. Kunis G. Ziv Y. Avidan H. Greenberg N.. (2006). Induction and blockage of oligodendrogenesis by differently activated microglia in an animal model of multiple sclerosis. J. Clin. Invest. 116, 905–915. 10.1172/jci26836 16557302 PMC1409740

[B16] Colton C. A. (2009). Heterogeneity of microglial activation in the innate immune response in the brain. J. Neuroimmune Pharmacol. 4, 399–418. 10.1007/s11481-009-9164-4 19655259 PMC2773116

[B17] Confavreux C. Vukusic S. (2006). Age at disability milestones in multiple sclerosis. Brain 129, 595–605. 10.1093/brain/awh714 16415309

[B18] Couillard-Despres S. Winner B. Schaubeck S. Aigner R. Vroemen M. Weidner N.. (2005). Doublecortin expression levels in adult brain reflect neurogenesis. Eur. J. Neurosci. 21, 1–14. 10.1111/j.1460-9568.2004.03813.x 15654838

[B19] Dutta R. Chang A. Doud M. K. Kidd G. J. Ribaudo M. V. Young E. A.. (2011). Demyelination causes synaptic alterations in hippocampi from multiple sclerosis patients. Ann. Neurol. 69, 445–454. 10.1002/ana.22337 21446020 PMC3073544

[B20] Fulci G. Dmitrieva N. Gianni D. Fontana E. J. Pan X. Lu Y.. (2007). Depletion of peripheral macrophages and brain microglia increases brain tumor titers of oncolytic viruses. Cancer Res. 67, 9398–9406. 10.1158/0008-5472.can-07-1063 17909049 PMC2850558

[B21] Geurts J. J. Bo L. Roosendaal S. D. Hazes T. Daniels R. Barkhof F.. (2007). Extensive hippocampal demyelination in multiple sclerosis. J. Neuropathol. Exp. Neurol. 66, 819–827. 10.1097/nen.0b013e3181461f54 17805012

[B22] Goldberg J. Clarner T. Beyer C. Kipp M. (2015). Anatomical distribution of cuprizone-induced lesions in C57BL6 mice. J. Mol. Neurosci. 57, 166–175. 10.1007/s12031-015-0595-5 26067430

[B23] Gordon S. Hamann J. Lin H. H. Stacey M. (2011). F4/80 and the related adhesion-GPCRs. Eur. J. Immunol. 41, 2472–2476. 10.1002/eji.201141715 21952799

[B24] Hart A. D. Wyttenbach A. Perry V. H. Teeling J. L. (2012). Age related changes in microglial phenotype vary between CNS regions: grey versus white matter differences. Brain Behav. Immun. 26, 754–765. 10.1016/j.bbi.2011.11.006 22155499 PMC3381227

[B25] Hefendehl J. K. Neher J. J. Sühs R. B. Kohsaka S. Skodras A. Jucker M. (2014). Homeostatic and injury-induced microglia behavior in the aging brain. Aging Cell 13, 60–69. 10.1111/acel.12149 23953759 PMC4326865

[B26] Hoffmann K. Lindner M. Gröticke I. Stangel M. Löscher W. (2008). Epileptic seizures and hippocampal damage after cuprizone-induced demyelination in C57BL/6 mice. Exp. Neurol. 210, 308–321. 10.1016/j.expneurol.2007.11.005 18096162

[B27] Hulst H. E. Schoonheim M. M. Van Geest Q. Uitdehaag B. M. J. Barkhof F. Geurts J. J. G. (2015). Memory impairment in multiple sclerosis: relevance of hippocampal activation and hippocampal connectivity. Mult. Scler. 21, 1705–1712. 10.1177/1352458514567727 25680986

[B28] Imai Y. Ibata I. Ito D. Ohsawa K. Kohsaka S. (1996). A novel gene iba1 in the major histocompatibility complex class III region encoding an EF hand protein expressed in a monocytic lineage. Biochem. Biophys. Res. Commun. 224, 855–862. 10.1006/bbrc.1996.1112 8713135

[B29] Ito D. Imai Y. Ohsawa K. Nakajima K. Fukuuchi Y. Kohsaka S. (1998). Microglia-specific localisation of a novel calcium binding protein, Iba1. Mol. Brain Res. 57, 1–9. 10.1016/s0169-328x(98)00040-0 9630473

[B30] Ito D. Tanaka K. Suzuki S. Dembo T. Fukuuchi Y. (2001). Enhanced expression of Iba1, ionized calcium-binding adapter molecule 1, after transient focal cerebral ischemia in rat brain. Stroke 32, 1208–1215. 10.1161/01.str.32.5.1208 11340235

[B31] Jack C. Ruffini F. Bar-Or A. Antel J. P. (2005). Microglia and multiple sclerosis. J. Neurosci. Res. 81, 363–373. 10.1002/jnr.20482 15948188

[B32] Jacobs A. H. Tavitian B. INMiND consortium. (2012). Noninvasive molecular imaging of neuroinflammation. J. Cereb. Blood Flow Metab. 32, 1393–1415. 10.1038/jcbfm.2012.53 22549622 PMC3390799

[B33] Kandasamy M. Lehner B. Kraus S. Sander P. R. Marschallinger J. Rivera F. J.. (2014). TGF-beta signalling in the adult neurogenic niche promotes stem cell quiescence as well as generation of new neurons. J. Cell. Mol. Med. 18, 1444–1459. 10.1111/jcmm.12298 24779367 PMC4124027

[B34] Kipp M. Clarner T. Dang J. Copray S. Beyer C. (2009). The cuprizone animal model: new insights into an old story. Acta Neuropathol. 118, 723–736. 10.1007/s00401-009-0591-3 19763593

[B35] Koutsoudaki P. N. Skripuletz T. Gudi V. Moharregh-Khiabani D. Hildebrandt H. Trebst C.. (2009). Demyelination of the hippocampus is prominent in the cuprizone model. Neurosci. Lett. 451, 83–88. 10.1016/j.neulet.2008.11.058 19084049

[B36] Le Blon D. Guglielmetti C. Hoornaert C. Quarta A. Daans J. Dooley D.. (2016). Intracerebral transplantation of interleukin 13-producing mesenchymal stem cells limits microgliosis, oligodendrocyte loss and demyelination in the cuprizone mouse model. J. Neuroinflammation 13:288. 10.1186/s12974-016-0756-7 27829467 PMC5103449

[B37] Lee D. C. Ruiz C. R. Lebson L. Selenica M. L. Rizer J. Hunt J. B. Jr.. (2013). Aging enhances classical activation but mitigates alternative activation in the central nervous system. Neurobiol. Aging 34, 1610–1620. 10.1016/j.neurobiolaging.2012.12.014 23481567 PMC3652232

[B38] Mallucci G. Peruzzotti-Jametti L. Bernstock J. D. Pluchino S. (2015). The role of immune cells, glia and neurons in white and gray matter pathology in multiple sclerosis. Prog. Neurobiol. 127–128, 1–22. 10.1016/j.pneurobio.2015.02.003 25802011 PMC4578232

[B39] Manouchehrinia A. Westerlind H. Kingwell E. Zhu F. Carruthers R. Ramanujam R.. (2017). Age related multiple sclerosis severity score: disability ranked by age. Mult. Scler. 23, 1938–1946. 10.1177/1352458517690618 28155580 PMC5700773

[B40] Martinez F. O. Gordon S. (2014). The M1 and M2 paradigm of macrophage activation: time for reassessment. F1000Prime Rep. 6:13. 10.12703/p6-13 24669294 PMC3944738

[B41] Matsushima G. K. Morell P. (2001). The neurotoxicant, cuprizone, as a model to study demyelination and remyelination in the central nervous system. Brain Pathol. 11, 107–116. 10.1111/j.1750-3639.2001.tb00385.x 11145196 PMC8098267

[B42] McMurran C. E. Jones C. A. Fitzgerald D. C. Franklin R. J. (2016). CNS remyelination and the innate immune system. Front. Cell Dev. Biol. 4:38. 10.3389/fcell.2016.00038 27200350 PMC4853384

[B43] Mildner A. Huang H. Radke J. Stenzel W. Priller J. (2017). P2Y12 receptor is expressed on human microglia under physiological conditions throughout development and is sensitive to neuroinflammatory diseases. Glia 65, 375–387. 10.1002/glia.23097 27862351

[B45] Miron V. E. (2017). Microglia-driven regulation of oligodendrocyte lineage cells, myelination, and remyelination. J. Leukoc. Biol. 101, 1103–1108. 10.1189/jlb.3ri1116-494r 28250011

[B44] Miron V. E. Boyd A. Zhao J. W. Yuen T. J. Ruckh J. M. Shadrach J. L.. (2013). M2 microglia and macrophages drive oligodendrocyte differentiation during CNS remyelination. Nat. Neurosci. 16, 1211–1218. 10.1038/nn.3469 23872599 PMC3977045

[B46] Moore C. S. Ase A. R. Kinsara A. Rao V. T. Michell-Robinson M. Leong S. Y.. (2015). P2Y12 expression and function in alternatively activated human microglia. Neurol. Neuroimmunol. Neuroinflamm. 2:e80. 10.1212/NXI.0000000000000080 25821842 PMC4370387

[B47] Moraga A. Pradillo J. M. García-Culebras A. Palma-Tortosa S. Ballesteros I. Hernández-Jiménez M.. (2015). Aging increases microglial proliferation, delays cell migration and decreases cortical neurogenesis after focal cerebral ischemia. J. Neuroinflammation 12:87. 10.1186/s12974-015-0314-8 25958332 PMC4437744

[B48] Mori I. Imai Y. Kohsaka S. Kimura Y. (2000). Upregulated expression of Iba1 molecules in the central nervous system of mice in response to neurovirulent influenza A virus infection. Microbiol. Immunol. 44, 729–735. 10.1111/j.1348-0421.2000.tb02556.x 11021405

[B49] Mosher K. I. Wyss-Coray T. (2014). Microglial dysfunction in brain aging and Alzheimer’s disease. Biochem. Pharmacol. 88, 594–604. 10.1016/j.bcp.2014.01.008 24445162 PMC3972294

[B50] Napoli I. Neumann H. (2010). Protective effects of microglia in multiple sclerosis. Exp. Neurol. 225, 24–28. 10.1016/j.expneurol.2009.04.024 19409897

[B51] Neumann H. Kotter M. R. Franklin R. J. (2009). Debris clearance by microglia: an essential link between degeneration and regeneration. Brain 132, 288–295. 10.1093/brain/awn109 18567623 PMC2640215

[B52] Norden D. M. Godbout J. P. (2013). Review: microglia of the aged brain: primed to be activated and resistant to regulation. Neuropathol. Appl. Neurobiol. 39, 19–34. 10.1111/j.1365-2990.2012.01306.x 23039106 PMC3553257

[B53] Norden D. M. Muccigrosso M. M. Godbout J. P. (2015). Microglial priming and enhanced reactivity to secondary insult in aging and traumatic CNS injury and neurodegenerative disease. Neuropharmacology 96, 29–41. 10.1016/j.neuropharm.2014.10.028 25445485 PMC4430467

[B54] Norkute A. Hieble A. Braun A. Johann S. Clarner T. Baumgartner W.. (2009). Cuprizone treatment induces demyelination and astrocytosis in the mouse hippocampus. J. Neurosci. Res. 87, 1343–1355. 10.1002/jnr.21946 19021291

[B55] Olah M. Amor S. Brouwer N. Vinet J. Eggen B. Biber K.. (2012). Identification of a microglia phenotype supportive of remyelination. Glia 60, 306–321. 10.1002/glia.21266 22072381

[B56] O’Loughlin E. Madore C. Lassmann H. Butovsky O. (2018). Microglial phenotypes and functions in multiple sclerosis. Cold Spring Harb. Perspect. Med. 8:a028993. 10.1101/cshperspect.a028993 29419406 PMC5793738

[B57] Perry V. H. Holmes C. (2014). Microglial priming in neurodegenerative disease. Nat. Rev. Neurol. 10, 217–224. 10.1038/nrneurol.2014.38 24638131

[B58] Pieper C. Marek J. J. Unterberg M. Schwerdtle T. Galla H. J. (2014). Brain capillary pericytes contribute to the immune defense in response to cytokines or LPS *in vitro*. Brain Res. 1550, 1–8. 10.1016/j.brainres.2014.01.004 24418464

[B59] Planche V. Koubiyr I. Romero J. E. Manjon J. V. Coupé P. Deloire M.. (2018). Regional hippocampal vulnerability in early multiple sclerosis: dynamic pathological spreading from dentate gyrus to CA1. Hum. Brain Mapp. 39, 1814–1824. 10.1002/hbm.23970 29331060 PMC6866595

[B60] Planche V. Ruet A. Coupe P. Lamargue-Hamel D. Deloire M. Pereira B.. (2017). Hippocampal microstructural damage correlates with memory impairment in clinically isolated syndrome suggestive of multiple sclerosis. Mult. Scler. 23, 1214–1224. 10.1177/1352458516675750 27780913

[B61] Praet J. Guglielmetti C. Berneman Z. Van der Linden A. Ponsaerts P. (2014). Cellular and molecular neuropathology of the cuprizone mouse model: clinical relevance for multiple sclerosis. Neurosci. Biobehav. Rev. 47, 485–505. 10.1016/j.neubiorev.2014.10.004 25445182

[B62] Ransohoff R. M. (2016). A polarizing question: do M1 and M2 microglia exist? Nat. Neurosci. 19, 987–991. 10.1038/nn.4338 27459405

[B63] Rawji K. S. Kappen J. Tang W. Teo W. Plemel J. R. Stys P. K.. (2018). Deficient surveillance and phagocytic activity of myeloid cells within demyelinated lesions in aging mice visualized by *ex vivo* live multiphoton imaging. J. Neurosci. 38, 1973–1988. 10.1523/jneurosci.2341-17.2018 29363580 PMC6705888

[B64] Rawji K. S. Yong V. W. (2013). The benefits and detriments of macrophages/microglia in models of multiple sclerosis. Clin. Dev. Immunol. 2013:948976. 10.1155/2013/948976 23840244 PMC3694375

[B65] Régnier-Vigouroux A. (2003). The mannose receptor in the brain. Int. Rev. Cytol. 226, 321–342. 10.1016/s0074-7696(03)01006-4 12921240

[B66] Sasaki Y. Hoshi M. Akazawa C. Nakamura Y. Tsuzuki H. Inoue K.. (2003). Selective expression of Gi/o-coupled ATP receptor P2Y12 in microglia in rat brain. Glia 44, 242–250. 10.1002/glia.10293 14603465

[B67] Schuitemaker A. van der Doef T. F. Boellaard R. van der Flier W. M. Yaqub M. Windhorst A. D.. (2012). Microglial activation in healthy aging. Neurobiol. Aging 33, 1067–1072. 10.1016/j.neurobiolaging.2010.09.016 21051106

[B68] Sicotte N. L. Kern K. C. Giesser B. S. Arshanapalli A. Schultz A. Montag M.. (2008). Regional hippocampal atrophy in multiple sclerosis. Brain 131, 1134–1141. 10.1093/brain/awn030 18375977

[B69] Sierra A. Abiega O. Shahraz A. Neumann H. (2013). Janus-faced microglia: beneficial and detrimental consequences of microglial phagocytosis. Front. Cell. Neurosci. 7:6. 10.3389/fncel.2013.00006 23386811 PMC3558702

[B70] Sonar S. A. Lal G. (2017). Differentiation and transmigration of CD4 T cells in neuroinflammation and autoimmunity. Front. Immunol. 8:1695. 10.3389/fimmu.2017.01695 29238350 PMC5712560

[B71] Steelman A. J. Thompson J. P. Li J. (2012). Demyelination and remyelination in anatomically distinct regions of the corpus callosum following cuprizone intoxication. Neurosci. Res. 72, 32–42. 10.1016/j.neures.2011.10.002 22015947 PMC3230728

[B72] Streit W. J. Xue Q. S. Tischer J. Bechmann I. (2014). Microglial pathology. Acta Neuropathol. Commun. 2:142. 10.1186/s40478-014-0142-6 25257319 PMC4180960

[B73] Sun J. Zhou H. Bai F. Ren Q. Zhang Z. (2016). Myelin injury induces axonal transport impairment but not AD-like pathology in the hippocampus of cuprizone-fed mice. Oncotarget 7, 30003–30017. 10.18632/oncotarget.8981 27129150 PMC5058659

[B74] Tagge I. O’Connor A. Chaudhary P. Pollaro J. Berlow Y. Chalupsky M.. (2016). Spatio-temporal patterns of demyelination and remyelination in the cuprizone mouse model. PLoS One 11:e0152480. 10.1371/journal.pone.0152480 27054832 PMC4824475

[B75] Thomsen J. H. Etzerodt A. Svendsen P. Moestrup S. K. (2013). The haptoglobin-CD163-heme oxygenase-1 pathway for hemoglobin scavenging. Oxid. Med. Cell. Longev. 2013:523652. 10.1155/2013/523652 23781295 PMC3678498

[B76] van Rossum D. Hilbert S. Strassenburg S. Hanisch U. K. Brück W. (2008). Myelin-phagocytosing macrophages in isolated sciatic and optic nerves reveal a unique reactive phenotype. Glia 56, 271–283. 10.1002/glia.20611 18069669

[B77] Zöller T. Attaai A. Potru P. S. Ruß T. Spittau B. (2018). Aged mouse cortical microglia display an activation profile suggesting immunotolerogenic functions. Int. J. Mol. Sci. 19:E706. 10.3390/ijms19030706 29494550 PMC5877567

[B78] Zrzavy T. Machado-Santos J. Christine S. Baumgartner C. Weiner H. L. Butovsky O.. (2017). Dominant role of microglial and macrophage innate immune responses in human ischemic infarcts. Brain Pathol. [Epub ahead of print]. 10.1111/bpa.12583 29222823 PMC6334527

