# Supplementary figures and images for: Age Influences Microglial Activation After Cuprizone-Induced Demyelination
Source: Front Aging Neurosci. 2018 Sep 20;10:278. doi: 10.3389/fnagi.2018.00278 (PMC6160739; doi:10.3389/fnagi.2018.00278)

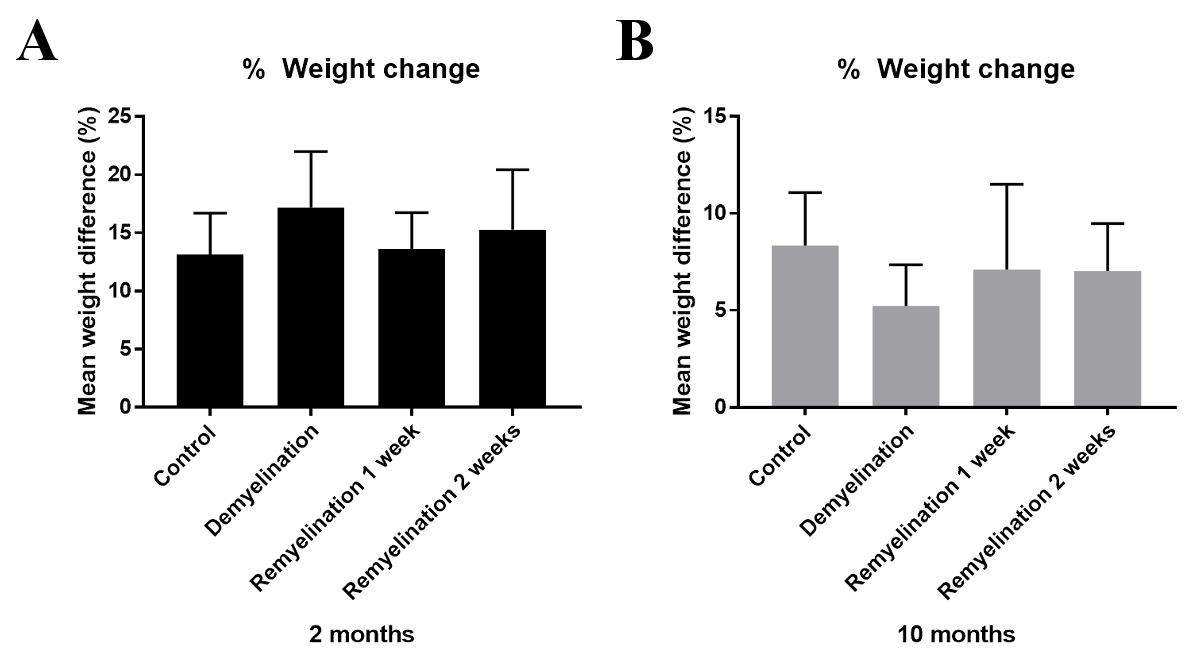

Supplement: FIGURE S1 — Weight changes during the experiment. Percentage weight changes between the first and the last 2 days of the experiment in (A) young and (B) middle-aged mice. In both age groups, no significant differences were observed in relative weight changes in comparison to the respective controls. Values are shown as means + SD (n = 6 per group). Statistical significance was evaluated using a one-way ANOVA and a Tukey’s post hoc test (A) and a Kruskal-Wallis one-way analysis followed by a Dunn’s multiple comparisons test (B). [file Image_1.jpg]
